# LONGITUDINAL ASSOCIATIONS BETWEEN TIME PERSPECTIVE AND LIFE SATISFACTION ACROSS ADULTHOOD

**DOI:** 10.1093/geroni/igae098.0373

**Published:** 2024-12-31

**Authors:** Maria Wirth, Markus Wettstein, Klaus Rothermund

**Affiliations:** Friedrich Schiller University Jena, Jena, Thuringen, Germany; Humboldt University Berlin, Berlin, Berlin, Germany; Friedrich Schiller University Jena, Jena, Thuringen, Germany

## Abstract

Subjective representations of time are an important predictor of well-being. How time is structured and represented in individual behavior and experience, is itself subject to developmental change. Young adults focus on preparing for their future, whereas during midlife, shifts towards a more present-oriented time perspective should arise. Realizing that important life goals may no longer be achievable should lead older adults to detach themselves from these goals and focus more on present and past experiences. These age-related changes in time perspective supposedly are associated with greater well-being. Previous studies, however, yielded heterogeneous results. However, these studies mostly investigated only one dimension of time perspective and did not include younger and/or middle-aged adults. To overcome these limitations, we investigated how changes in four facets of time perspective (orientation towards the past, concreteness of the future, obsolescence, and finitude of life) are related to changes in life satisfaction and whether these associations are moderated by chronological age. We used data from an age-diverse sample comprising 459 participants (30 – 80 years) of the “Aging as Future” (AAF) study comprising three measurement occasion over 10 years. Longitudinal multilevel regressions indicated that individuals with overall higher concreteness and lower obsolescence reported higher life satisfaction. Additionally, individuals reported higher life satisfaction on measurement occasions when they scored higher on concreteness. These associations were not moderated by age, which questions the idea of age-differential associations between time perspective and well-being. Our study provides a deeper understanding of the relation between time perspective and well-being across adulthood.

